# Development and validation of an improved prediction model for vaginal birth after previous cesarean section: a retrospective cohort study of a Chinese population

**DOI:** 10.1080/07853890.2025.2523617

**Published:** 2025-06-28

**Authors:** Haiyan Liu, Yi Yu, Xiaoyue Zhang, Jiangnan Pei, Yao Tang, Rong Hu, Weirong Gu

**Affiliations:** Department of Obstetrics, Obstetrics and Gynecology Hospital of Fudan University, Shanghai, China

**Keywords:** Vaginal birth after cesarean, prediction model, temporal validation, ratio of weight gain to pre-pregnancy weight, interval time of pregnancies, Chinese population

## Abstract

**Objective:**

The transition from one-child to two-child and three-child policy in China has increasingly led to a rise in the number of women who choose trial of labor after cesarean section (TOLAC). Achieving vaginal birth after cesarean section (VBAC) is, however, not always guaranteed, and a failed TOLAC is associated with a high risk of maternal and neonatal complications. Although Grobman’s model may help predict VBAC, variations in population characteristics and healthcare settings can limit its generalizability and validity on a global scale. This study, therefore, seeks to develop and validate an improved prediction model for VBAC at the onset of labor among the Chinese population.

**Methods:**

Seven hundred and twenty women who attempted a TOLAC were enrolled. The development dataset comprised 481 women, while the other 239 women constituted the temporal validation dataset. Variable selection was executed using the least absolute shrinkage and selection operator method. Model development was performed using logistic regression techniques and was presented as a nomogram.

**Results:**

Of the participants, 81.4% achieved VBAC. The model included maternal age, maternal height, ratio of weight gain to pre-pregnancy weight, interval time of pregnancies, previous vaginal delivery, premature rupture of membranes, oxytocin administration, spontaneous labor onset, labor analgesia, and newborn weight. The development and temporally validated areas under the curve were 0.780 (95% confidence interval 0.726–0.834) and 0.774 (95% confidence interval 0.694–0.854), respectively. Internal validation performed by bootstrap resampling, calibration curves, and Hosmer-Lemeshow test confirmed the model’s robust performance. An optimal predicted probability cut-off of 0.7 was identified by decision curve analysis and clinical considerations.

**Conclusions:**

The improved predictive VBAC model exhibited adequate performance such that women with a prior low transverse cesarean delivery who scored 0.7 or higher (in the model-derived probability score) would consider TOLAC, potentially leading to a reduction in maternal-neonatal morbidity.

**Registration:**

The study was approved by the Ethical Committee of Obstetrics and Gynecology Hospital, Fudan University (2018-43) and was registered in the Chinese Clinical Trial Registry (ChiCTR1900022484), https://www.chictr.org.cn/showproj.html?proj=37898. The study adhered to the Declaration of Helsinki. The first participant was enrolled on January 1, 2016. The requirement for informed consent was waived because the data were anonymized.

## KEY MESSAGES

- An improved prediction model that underwent both internal and temporal validation performed well for the Chinese VBAC in the adjustment of China’s fertility policy setting.
- Pregnancy interval of more than 10 years became an unfavorable factor for VBAC.
- Predictors of the ratio of weight gain to pre-pregnancy weight marked a pivotal advancement in acknowledging the impact of pre-pregnancy obesity and judicious gestational weight management on the likelihood of achieving a VBAC.

## 1. Introduction

The escalating prevalence of cesarean section (CS) in recent decades represents a significant global concern, a trend notably observed in China [1,2]. The introduction of the two-child policy in 2016 and its subsequent transition to a three-child policy in 2021 has led to an increasing number of women who have previously undergone CS opting for subsequent pregnancies in China [3,4]. Consequently, obstetricians and women who have experienced prior CS deliveries face intricate decision-making processes concerning the most appropriate mode of delivery for subsequent pregnancies.

In addition to elective repeated cesarean section, the option of trial of labor after cesarean section (TOLAC) stands as an alternative mode of delivery, potentially resulting in either successful TOLAC, known as vaginal birth after cesarean (VBAC), or emergency CS (failed TOLAC). Among women with previous CS, the rates of maternal and neonatal complications were observed to be the lowest among those who achieved VBAC, while the highest rates were noted in cases of failed TOLAC [1]. Evidently, when counselling on the mode of delivery for women with previous CS, the probability of achieving VBAC is a pivotal component. The reported success rates for TOLAC range between 60% and 80% worldwide [5]. However, it is important to note that these rates serve as general benchmarks and may not be universally applicable for individualized counselling owing to diverse demographic and obstetric characteristics. Therefore, the establishment of a personalized prediction model for VBAC could contribute to the reduction of maternal-neonatal morbidity.

Research on VBAC screening tools that undergo both internal and temporal validations is scarce, particularly in the context of the Chinese population. Grobman’s enhanced predictive model, utilizing information available during the first prenatal visit (maternal age, height, pre-pregnancy weight, arrest indication for previous cesarean section, previous vaginal delivery only before prior cesarean, previous VBAC, and treated chronic hypertension) and excluding race and ethnicity as factor [6], appears to be applicable for estimating the likelihood of achieving VBAC in the Chinese population. However, it is crucial to acknowledge that variations in population characteristics and healthcare settings within Asian countries such as China may impact the generalizability and validity of the prediction model. For instance, in the United States, TOLAC has been a standard approach for over 40 years, with fluctuations in attempted TOLAC rates from 30.2% in 1996 to 11.3% in 2003 and subsequently rising to 21.7% by 2020 [7]. By contrast, the promotion of TOLAC in China began in 2010, accompanied by the issuance of a consensus on VBAC management in 2016 [8]. The implementation of TOLAC in China, set against the backdrop of strained doctor-patient relationships, is limited to a select number of medical centers, where the attempted TOLAC rate is approximately 10% [9–11]. Moreover, Grobman’s enhanced model does not consider intrapartum variables, such as labor induction and estimated fetal weight. Consequently, our study aimed to develop and temporally validate an improved prediction model tailored for VBAC at the onset of labor in the Chinese population.

## 2. Material and methods

### 2.1. Data sources

This retrospective cohort study was conducted from January 1, 2016, to December 31, 2022, at our center. Functioning as a tertiary academic medical center, the hospital boasts continuous 24-hour in-house obstetric and anesthetic coverage. With an annual delivery volume of approximately 12,000, the institution plays a pivotal role in delivering healthcare to the obstetrical population within and surrounding the region, particularly those classified as high-risk. Because the data were anonymized, the informed consent requirement was waived by the Ethical Committee of Obstetrics and Gynecology Hospital, Fudan University.

Eligible candidates for TOLAC, those admitted, based on the obstetricians’ assessments, met the following inclusion criteria: (1) age ≥18 years and <50 years with TOLAC intention recorded in the prenatal visit record, (2) women who had a previous cesarean delivery with a low-transverse incision, (3) interval time of pregnancies (the duration between the last menstrual period of the current pregnancy and the previous CS) ≥18 months, (4) cephalic presentation of a single fetus and no previous indications for CS or new indications for CS, (5) continuous lower uterine segment scar shown by ultrasound examination in the late third trimester, and (6) gestational age at delivery between 37 + 0 and 41 + 6 weeks.

Exclusion criteria were as follows: (1) previous CS with a classical incision, T-shaped incision, unknown incision, or incision tear; (2) history of uterine scar pregnancy surgery, uterine rupture, or dehiscence; (3) multiple pregnancies, abnormal fetal position, or threatened uterine rupture or dehiscence; (4) presence of infection or uterine diverticulum after a previous CS; (5) history of myomectomy; and (6) unsuitability for vaginal delivery due to severe medical or surgical complications.

Data were obtained from electronic medical records. All data were audited periodically by trained technical personnel to ensure their validity, thereby mitigating any potential information bias. Prior to analysis, personal information pertaining to each participant was safeguarded through anonymization. The study was approved by the local institutional ethics committee (2018-43) and registered in the Chinese Clinical Trial Registry (ChiCTR1900022484) on April 13, 2019. This was a retrospective observational cohort study, then the first participant was enrolled on January 1, 2016. The requirement for informed consent was waived because the data were anonymized.

### 2.2. Variables of interest

We collected the following variables: maternal characteristics including maternal age at delivery, maternal height, interval time of pregnancies, weight gain (calculated as maternal weight at delivery in kilograms minus pre-pregnancy weight in kilograms), presence of diabetic and hypertensive disorders, any previous vaginal delivery, and maximum birth weight of the previous child; current delivery characteristics including gestational age at delivery, premature rupture of membranes (PROM), spontaneous labor onset, oxytocin administration (whether for labor induction or augmentation), labor analgesia, mode of delivery, and maternal and neonatal outcomes including postpartum hemorrhage, uterine rupture, hysterectomy, 3rd and 4th perineal tears, newborn weight, neonatal asphyxia, and neonatal intensive care unit admission. All uterine ruptures were observed during the laparotomy. A complete uterine rupture was defined as rupture of all layers of the uterine muscle and serosa, while incomplete uterine rupture (dehiscence) was characterized by preservation of the intact uterine serosa.

Based on the literature and clinical experience, we identified potential predictive factors for VBAC and selected the variables that were most likely to have good predictive abilities. We finally considered the following 12 candidate predictors: maternal age, maternal height, ratio of weight gain to pre-pregnancy weight, delivery week, newborn weight, ratio of newborn weight to the maximum birth weight of previous pregnancies, interval time of pregnancies, spontaneous labor onset, oxytocin administration, PROM, labor analgesia, and vaginal delivery history.

### 2.3. Sample size

The sample size for the development set was calculated according to the four-step procedure of Riley RD and the pmsamp size package in R [12]. Considering the 12 candidate predictor parameters, an assumption was made based on previously published data, positing that the rate of VBAC in our hospital would be 80% [9]. For an outcome proportion of 0.80, the maximum R^2^ value is 0.632. If we posit that the new model explains 50% of the variability, the anticipated R^2^ value is 0.5 × 0.632 = 0.316. Then, the initially calculated sample size for the development set was established for at least 309 women.

In accordance with the R package sampsizeval elucidated in the literature [13] and considering a TOLAC success rate of 80% in our center, the estimated area under the curve (AUC) value was 0.75, the anticipated standard error of AUC was 0.05, and the anticipated standard error of the slope and intercept of the calibration chart was 0.2, the sample size for the temporal validation set was calculated to be 145, 236, and 182 participants, respectively. The maximum value was selected, indicating that at least 236 women were required to participate.

### 2.4. Statistical analysis

The chi-squared test or Fisher’s exact test was used to evaluate categorical variables. For continuous variables, Student’s t-test was used for data with a normal distribution, while the Mann–Whitney U test was employed for data with skewed distribution. Statistical significance was set at *p* value < 0.05.

### 2.5. Variable selection and model construction

The development dataset was utilized for variable selection and prediction model development. To mitigate potential multicollinearity and overfitting among the predictive variables, the least absolute shrinkage and selection operator (LASSO) method was employed. LASSO shrinks the absolute size of the coefficients by applying penalties based on the tuning parameter λ [14,15]. With increasing penalties, the weaker factor coefficients approach zero, while the strongest predictors are retained, facilitating effective variable selection. Ten-fold cross-validation was implemented within the LASSO binary logistic regression to determine the optimal tuning parameter λ and minimize the average error. The ‘glmnet’ package in the R statistical environment was utilized to execute the LASSO regression model. The variables identified through the LASSO analysis were subsequently integrated into the final logistic regression model. These variables were then presented as nomograms to facilitate clinical application.

### 2.6. Model performance and validation

Mode discrimination was evaluated by calculating the AUC, while calibration was assessed using the calibration slope and Hosmer-Lemeshow test. The determination of a clinical cutoff point was undertaken through risk stratification analysis, and its clinical utility was further examined using a decision curve. Internal validation of the model was conducted through 500 bootstrap resamplings. Temporal validation was performed to assess the model’s reproducibility using a dataset from the same center, collected between June 1, 2020, and December 31, 2022. A complete case analysis strategy was employed, restricting the analysis to patients with non-missing data for all the candidate variables. All statistical analyses were performed using the R software (version 4.1.3, R Foundation, https://cran.r-project.org).

## 3. Results

### 3.1. General demographic and clinical characteristics of the development and temporal validation datasets

**Figure 1.**
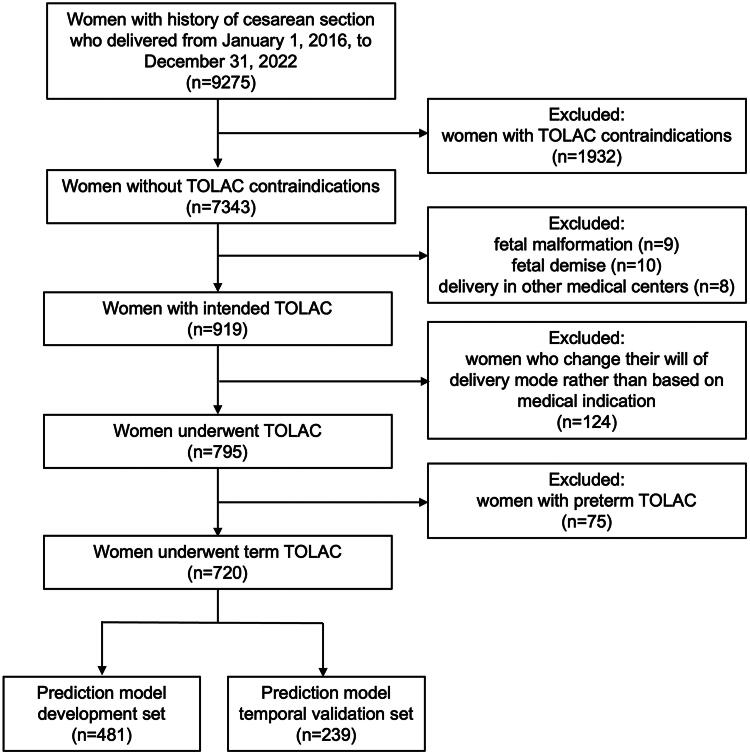
Patients flow chart.

During the observation period, 9,275 women with a history of CS were identified. Among these, 1,932 individuals with contraindications for TOLAC were excluded from the study. Additionally, 124 women who opted for CS during delivery without medical indications except for previous CS were excluded from the analysis. Ultimately, 720 pregnancies (9.8%, 720/7,324) meeting the study criteria, all of Chinese ethnicity, and at full term were included in the final analysis, as illustrated in Figure 1. Within the study cohort, the majority of 586 women (81.4%, 586/720) achieved VBAC. The annual cesarean delivery rate at our center was approximately 40% throughout the study period.

During the actual data collection period spanning from January 1, 2016, to May 31, 2020, a cohort of 481 women who underwent TOLAC was identified. Consequently, the final sample size for our development set was 481 women. For the actual temporal validation set, 239 women were enrolled between June 1, 2020, and December 31, 2022. Table 1 shows the differences in the demographic and clinical characteristics between the development and temporal validation datasets. Compared to women in the development set, women in the temporal validation set exhibited higher pre-pregnancy BMI and BMI at delivery, lower weight gain during pregnancy, elevated prevalence of gestational diabetes mellitus, longer interval time of pregnancies, increased incidences of cervical ripening and oxytocin administration, and reduced incidence of spontaneous labor onset.

**Table 1.** Demographic and clinical characteristics between development and validation datasets (*n* = 720).

Characteristics	Total cohort (*n* = 720)	Development dataset (*n* = 481)	Validation dataset (*n* = 239)	*p* value*
Maternal age at delivery, years	33.22 (3.94)	32.94 (4.09)	33.78 (3.55)	0.007
Maternal height, cm	161.57 (4.60)	161.74 (4.54)	161.24 (4.69)	0.173
Pre-pregnancy weight, kg	56.46 (7.66)	55.65 (7.41)	58.11 (7.90)	<0.001
Pbstetrical history				
Previous vaginal delivery only before previous CS, (%)	22 (3.1)	15 (3.1)	7 (2.9)	1
Previous VBAC, (%)	34 (4.7)	18 (3.7)	16 (6.7)	0.116
Any vaginal delivery history, (%)	56 (7.8)	33 (6.9)	23 (9.6)	0.206
Details of previous CS				
Arrest labor indication, (%)	64 (8.9)	38 (7.9)	26 (10.9)	0.237
Previous CS in labor, (%)	130 (18.1)	86 (17.9)	44 (18.4)	0.943
Weight gain, kg	13.58 (4.25)	13.98 (4.29)	12.77 (4.05)	<0.001
The ratio of weight gain to pre-pregnancy weight	0.24 (0.08)	0.25 (0.08)	0.22 (0.08)	<0.001
Interval time of pregnancies, month	72.07 (35.82)	70.01 (33.95)	76.20 (39.06)	0.029
Pre-pregnancy BMI, kg/m^2^	21.58 (2.66)	21.23 (2.55)	22.28 (2.74)	<0.001
BMI at delivery, kg/m^2^	26.79 (3.03)	26.57 (3.01)	27.24 (3.05)	0.005
Gestational age at delivery, weeks	39.38 (1.04)	39.36 (1.04)	39.41 (1.03)	0.585
Spontaneous labor onset, (%)	551 (76.5)	395 (82.1)	156 (65.3)	<0.001
Cervical ripening, (%)	106 (14.7)	56 (11.6)	50 (20.9)	0.001
Oxytocin administration, (%)	194 (26.9)	103 (21.4)	91 (38.1)	<0.001
PROM, (%)	173 (24.0)	106 (22.0)	67 (28.0)	0.093
Cervix Bishop score	6.25 (1.32)	6.37 (1.35)	6.01 (1.23)	0.001
Neonatal weight, kg	3.38 (0.37)	3.38 (0.37)	3.39 (0.37)	0.868
Ratio of newborn weights	1.02 (0.14)	1.02 (0.14)	1.02 (0.15)	0.958
Labor analgesia, (%)	408 (56.7)	260 (54.1)	148 (61.9)	0.054
Pregnancy complications				
Gestational diabetes/pre-pregnancy diabetes, (%)	86 (11.9)	45 (9.4)	41 (17.2)	0.004
Hypertensive disorders, (%)	15 (2.1)	9 (1.9)	6 (2.5)	0.773

VBAC, vaginal birth after cesarean; BMI, body mass index; PROM, premature rupture of membrane; CS, cesarean section. Ratio of newborn weights is defined as the newborn weight of present pregnancy compared with the maximum birthweight of previous pregnancies. Data are mean (standard deviation), or n (%). * Chi-square test, Fisher’s exact test or Student-t test as appropriate.

### 3.2. Maternal-neonatal outcomes

Within the total TOLAC cohort, 1.7% (12/720) of patients experienced uterine rupture, with 0.4% (3/720) encountering complete uterine rupture. Among these cases, one patient with complete uterine rupture had concurrent bladder injury and neonatal asphyxia. The VBAC group exhibited only one instance of postpartum uterine rupture (incomplete). Additionally, 4.9% (35/720) of the women undergoing TOLAC were diagnosed with postpartum hemorrhage, and 1.7% (12/720) required blood transfusions. Furthermore, 0.7% (5/720) of newborns experienced neonatal asphyxia. In terms of delivery outcomes (Table S1), failed TOLAC was associated with higher rates of adverse maternal and neonatal outcomes (uterine rupture, 1 min Apgar score ≤7, and neonatal asphyxia). Notably, the analysis revealed the absence of 3rd and 4th perineal tears, hysterectomies, and reported maternal and neonatal deaths.

### 3.3. Predictor selection and model construction

**Figure 2.**
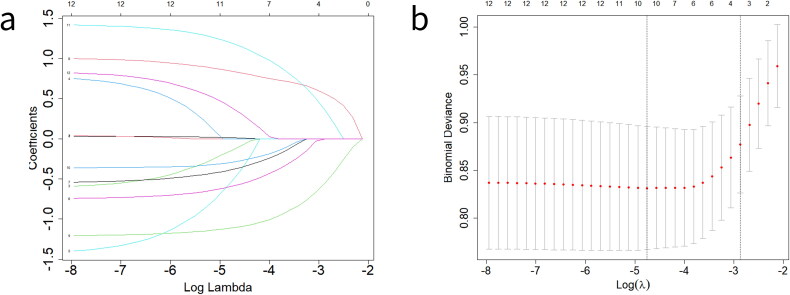
Variables selection by least absolute shrinkage and selection operator (LASSO). (a) variables selection by LASSO regression model with the coefficient profiles of the 12 variables. (b) Variables selection by LASSO regression used 10-fold cross-validation while the tuning parameter λ was selected in the minimal average error.

Twelve variables, as described above, were subjected to LASSO regression analysis, resulting in the identification of ten variables that retained significance as predictors of VBAC. These influential variables included maternal age at delivery ≥35 years, interval time of pregnancies > 10 years, ratio of weight gain to pre-pregnancy weight, maternal height, spontaneous labor onset, oxytocin administration, PROM, labor analgesia, vaginal delivery history, and newborn weight (Figure 2, Table S2).

**Figure 3.**
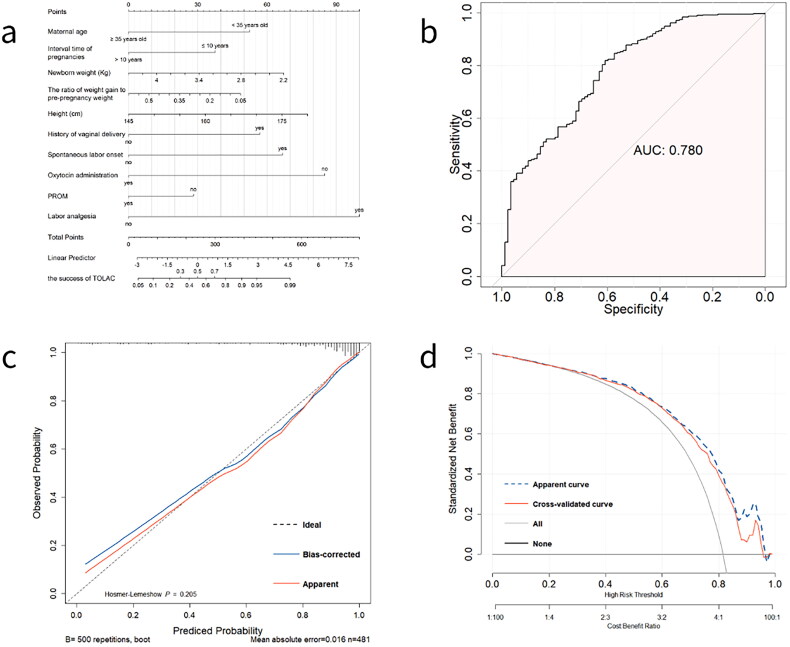
Nomogram, ROC plot, calibration plot and DCA plot of the model in development dataset. (a) a nomogram for the prediction of the likelihood of achieving vaginal birth after cesarean (VBAC). To use the nomogram, identify the position of every variable on its corresponding axis, draw a vertical line to the points axis to get the corresponding points of the variable, add all the points from every variable to the total points, and draw a vertical line from the total points axis to the axis representing ‘the success of TOLAC’ for the probability of achieving VBAC. (b) The area under the receiver-operator characteristic curve (ROC) of predicting successful VBAC among women with the trial of labor after cesarean section (TOLAC) in the development dataset. (c) Calibration plot with 500 bootstrap resamples for the internal validation to predict successful VBAC among women with TOLAC in the development dataset. The diagonal interrupted line is the line of ideal agreement. The red and the blue solid curves indicate a calibration curve using the loess nonparametric smoother and 500 bootstrap resamples, respectively. (d) Decision curve analysis (DCA) for the prediction model by 10-fold cross-validation in different thresholds in the development dataset. The red cross-validated curve indicated the average results was obtain from the 10-fold cross-validation and showed the robust estimates of the net benefit across all thresholds.

All ten predictors were fitted to the logistic model (Table 2): logit(P) = −2.4301 + (−0.7508; maternal age ≥ 35 years) + (−1.2616; ratio of weight gain to pre-pregnancy weight) + (0.0317; maternal height) + (−0.5368; interval time of pregnancies > 10 years) + (0.9550; spontaneous labor onset) + (−1.2157; oxytocin administration) + (−0.4023; PROM) + (1.4302; labor analgesia) + (0.8135; vaginal delivery history) + (−0.4365; newborn weight). The model was constructed and presented as a nomogram (Figure 3(a)).

**Table 2.** Multivariable logistic regression analysis of predictors for VBAC.

Variables	OR	95%CI	*p* value
Maternal age ≥ 35 years old	0.47	(0.27, 0.82)	0.001
Interval time of pregnancies > 10 years	0.58	(0.23, 1.55)	0.267
Spontaneous labor onset	2.60	(1.21, 5.56)	0.014
Oxytocin administration	0.30	(0.14, 0.63)	0.002
PROM	0.67	(0.37, 1.24)	0.194
Labor analgesia	4.18	(2.41, 7.48)	<0.001
Vaginal delivery history	2.26	(0.74, 8.79)	0.190
The ratio of weight gain to pre-pregnancy weight	0.28	(0.01, 6.60)	0.428
Maternal height	1.03	(0.97, 1.10)	0.308
Newborn weight	0.65	(0.31, 1.35)	0.246

VBAC, vaginal birth after cesarean; OR, odds ratio; CI, confidence interval; PROM, premature rupture of membranes.

### 3.4. Evaluation of model performance and validation

The AUC (Figure 3(b)) of the model was 0.780 (95% confidence interval [CI], 0.726–0.834). The robustness of these findings was further substantiated through internal validation using bootstrap resampling in the development dataset, yielding a mean AUC of 0.780 (95% CI, 0.741–0.852). To assess the calibration of the model, calibration curves were generated, as illustrated in Figure 3(c). These curves reveal an alignment between the predicted and observed probabilities of VBAC. The non-significant result of the Hosmer-Lemeshow test (X^2^ = 10.935, *p* = 0.205) also showed good calibration of the model.

The decision curve analysis presented in Figure 3(d) demonstrates the superior net benefit derived from employing the prediction model compared to scenarios where all or no women underwent intervention, particularly across the absolute risk threshold ranging from 0.3. While a universally agreed-upon threshold probability remains elusive, accumulating evidence suggests that women with at least a 60–70% likelihood of achieving VBAC encounter comparable or reduced maternal-neonatal morbidity compared to those opting for elective repeat cesarean delivery [16].

The detection performance of the model is summarized at various cut-off values (Table 3). Given the adverse maternal-neonatal outcomes associated with failed TOLAC, an optimal predicted-probability cut-off of 0.7 was identified. This threshold offers a balance between relatively high specificity, sensitivity, and positive predictive value. A predicted probability exceeding 0.7 designated women with an approximate success rate of nearly 90% (88.2%) for achieving VBAC. Simultaneously, women with a predicted probability below 0.7 were supposed to carry a 50% likelihood (50.6%) of undergoing failed TOLAC.

**Table 3.** The selection of risk threshold.

Predicted probability	Sensitivity	Specificity	PPV	NPV	Accuracy
0.5	0.98	0.315	0.863	0.778	0.857
0.6	0.946	0.382	0.871	0.618	0.842
0.7	0.895	0.472	0.882	0.506	0.817
0.75	0.849	0.551	0.893	0.454	0.794
0.8	0.755	0.629	0.9	0.368	0.732

PPV, positive predictive value; NPV, negative predictive value.

**Figure 4.**
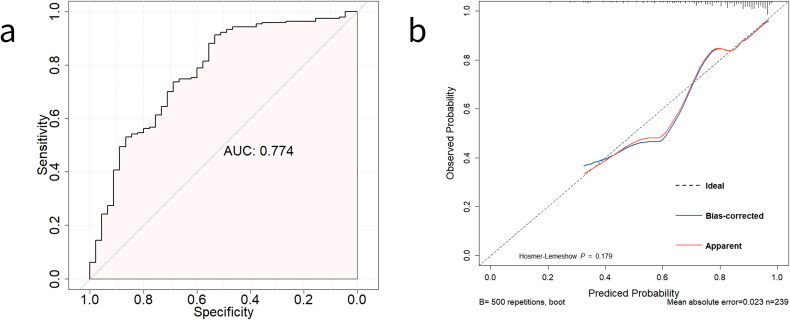
ROC Plot and calibration plot of prediction model for achieving VBAC in temporal validation dataset. (a) The area under the receiver-operator characteristic curve(ROC)of predicting successful vaginal birth after cesarean (VBAC) among women with the trial of labor after cesarean section (TOLAC) in the temporal validation dataset. (b) Calibration plot with 500 bootstrap resamples for the temporal validation to predict successful VBAC among women with TOLAC in the temporal validation dataset. The diagonal interrupted line is the line of ideal agreement. The red and the blue solid curves indicate a calibration curve using the loess nonparametric smoother and 500 bootstrap resamples, respectively.

Temporal validation of our prediction model confirmed its commendable performance. The model’s discriminative capacity was deemed reasonable, yielding an AUC of 0.774 (95% CI 0.694–0.854) (Figure 4(a)). The calibration analysis was represented by the slopes of the calibration curves and the nonsignificant Hosmer-Lemeshow test results (X^2^ = 11.415, *p* = 0.179) (Figure 4(b)). The overall calibration demonstrated acceptability; noteworthy excellence was observed within the range of high predicted probabilities of a VBAC greater than 70%. Meanwhile, most women fell within the high predicted probabilities of achieving a VBAC > 70%. When the cutoff value was set at 0.7, in the temporal validation dataset, 89.2% of those predicted to achieve VBAC could finally be successful, and 60% of women actually delivered by emergency cesarean section could be correctly identified (Table 4).

**Table 4.** Comparison of detection performance at various prediction value cut-offs for the VBAC prediction model in the temporal validation dataset.

Prediction value cut-off	Sensitivity	Specificity	PPV	NPV	Accuracy
0.5	0.943	0.444	0.88	0.645	0.849
0.6	0.907	0.533	0.893	0.571	0.837
0.7	0.768	0.600	0.892	0.375	0.736
0.75	0.727	0.689	0.910	0.369	0.72
0.8	0.655	0.711	0.907	0.323	0.665

PPV, positive predictive value; NPV, negative predictive value.

## 4. Discussion

This study developed and temporally validated a customized predictive model for VBAC at the onset of labor specifically tailored to the Chinese population who underwent a previous single low transverse cesarean delivery. The refined model that incorporated a comprehensive set of variables, including maternal age, maternal height, ratio of weight gain to pre-pregnancy weight (first proposed), interval time of pregnancies (first proposed), previous vaginal delivery, PROM, oxytocin administration, spontaneous labor onset, labor analgesia, and newborn weight, had a good prediction performance with an AUC of 0.780 (95% CI 0.726–0.834). In the context of our institution, we propose a practical threshold for decision-making, suggesting that individuals with a model-derived probability score of 0.7 or higher consider TOLAC. This recommendation underscores the critical importance of accurately selecting candidates for TOLAC to reduce maternal-neonatal morbidity.

TOLAC failure exacerbates numerous risks, including uterine rupture, postpartum hemorrhage, increased blood transfusion, and infant asphyxia or perinatal death [17]. Our study obtained similar results. In our cohort study, 11 of the 12 cases of uterine rupture (comprising 3 complete ruptures and 9 incomplete ruptures) were observed in the failed TOLAC group. Additionally, there were four cases of neonatal asphyxia in the failed TOLAC group and one case in the VBAC group. Owing to prompt detection and intervention, no instances of hysterectomy, maternal mortality, or neonatal mortality have been reported.

Consistent with previous studies [6,18–21], our investigation identified that advanced age and oxytocin administration emerged as negatively associated factors for VBAC. We also found significant positive effects of maternal height, labor analgesia, and spontaneous labor onset on achieving VBAC, consistent with the existing literature [6,18,21–24].

In contrast to models developed in regions with a longstanding history of TOLAC implementation and higher TOLAC rates, such as the United States [6,18,19], the weight assigned to previous vaginal delivery in our equation was relatively small, reflecting the unique clinical context of our cohort. Previous VBAC and vaginal delivery have been recognized as independent factors for achieving VBAC [25,26]. However, the findings of our study, employing LASSO regression analysis, did not reveal a statistically significant association between the likelihood of VBAC and a history of previous VBAC. A plausible explanation for this incongruity is the limited representation of women with a history of VBAC in our cohort, which constituted only 4.7% of the study population. TOLAC was implemented relatively late in China, and the rate of the intended term TOLAC remained low at 9.8% in our center. Given the infrequent occurrence of previous VBAC in our cohort, we amalgamated women with previous vaginal delivery both before and after CS into a unified variable termed ‘previous vaginal delivery’. In our prediction model, the coefficient assigned to a previous vaginal delivery was 0.8135, underscoring its substantial predictive capacity for VBAC.

Maternal obesity has consistently been identified as an unfavorable factor for achieving VBAC [19,27,28]. The management of weight gain during pregnancy, tailored to the individual’s pre-pregnancy BMI, has emerged as a pivotal element in controlling fetal weight and fostering conditions conducive to successful vaginal labor, including VBAC [29]. Our model identified the contributing variable, ratio of weight gain to pre-pregnancy weight, as an additional unfavorable predictor of VBAC for the first time. This predictive factor simultaneously considers the impact of obesity before and during pregnancy on the probability of VBAC. Therefore, the predictor had a more pronounced effect of maternal obesity on the likelihood of achieving VBAC than simpler factors, such as pre-pregnancy weight, pre-pregnancy BMI, and BMI before delivery. This marked a pivotal advancement in acknowledging the impact of pre-pregnancy obesity and judicious gestational weight management on the likelihood of achieving a VBAC. A recent study from China disclosed that excessive weight gain after 28 weeks of gestational age decreased the possibility of achieving VBAC, irrespective of pre-pregnancy BMI and weight gain before 28 weeks [30]. Unlike most other factors, gestational weight gain, a potential risk factor for obesity, can be modified during pregnancy. Therefore, obesity should not be considered as an absolute contraindication for TOLAC. For obese women intending TOLAC, reasonable weight management during pregnancy, especially after 28 weeks of gestational age, may improve the likelihood of VBAC.

Moreover, our study revealed that a pregnancy interval of > 10 years was an unfavorable factor for VBAC. The evolving landscape of China’s birth policy has witnessed a trend toward an increase in second and even third births, contributing to a higher prevalence of pregnant women with a pregnancy interval exceeding 10 years. This demographic shift has implications for the VBAC in our population. Rao et al. reported that an inter-delivery interval of 120 months or more increased the risk of major maternal and neonatal outcomes for TOLAC in Chinese population [31]. Mesay et al. revealed that VBAC was over six times more likely when the inter-delivery gap was beyond two years in Northwest Ethiopia, a country with a high intended TOLAC rate [32]. This indicates that a short pregnancy interval reduces the VBAC rate. Further studies are needed to determine the appropriate pregnancy interval for VBAC.

In our analysis, we observed that TOLAC women with ruptured membranes exhibited a decreased likelihood of achieving VBAC, a finding that deviated from the results reported in other studies abroad [32,33]. However, the results of Li ‘s study on the Chinese TOLAC cohort were consistent with ours [34]. One plausible explanation for this discrepancy may be rooted in the different clinical management strategies used for women with PROM. In our center, if labor failed to initiate within 2–12 h of membrane rupture, a low dose of intravenous oxytocin was administered to induce labor in line with the Chinese guidelines for the diagnosis and management of PROM in 2015 [35]. However, according to the American College of Obstetricians and Gynecologists Practice Bulletin Number 217, nearly 80% and 95% of women with PROM at term start labor spontaneously within 12 h and 24 h, respectively. A period of 12–24 h of expectant management is reasonable as long as the clinical and fetal conditions are reassuring [36]. Spontaneous labor onset is known to be a favorable factor for VBAC, whereas oxytocin administration is an unfavorable factor. These different clinical management practices may have led to different results.

Previous studies generally agree that macrosomia is a risk factor for TOLAC failure [37,38]. Similarly, we found that high newborn weight was an unfavorable predictor of VBAC. Our model utilized newborn weight as a potential predictor, as opposed to estimated fetal weight, limiting the applicability of the data for antenatal decision-making regarding delivery mode. Furthermore, we analyzed the ratio of current newborn weight to maximum previous birth weight. However, this indicator has not been integrated into the model. We posited that despite the absence of a formal prediction model in the counselling of delivery mode, the TOLAC population might have been somewhat pre-selected. Nonetheless, it remains appropriate for obstetricians and patients to weigh factors, such as previous birth weight and current estimated fetal weight, when making decisions regarding TOLAC.

Our study did not find a correlation between maternal complications, specifically diabetic and hyper­tensive disorders, and the likelihood of achieving VBAC. However, the current literature on this topic is inconsistent. Several researchers believe that diabetic and hypertensive disorders would lead to TOLAC failure [24,39]. Grobman’s improved model found that VBAC was significantly less likely in women with a history of medication-treated chronic hypertension [6]. Nonetheless, our findings are consistent with those of certain studies that reported results contrary to this perspective [18,19,40]. Several factors contribute to the observed disparities. First, the variation in the prevalence of diabetic and hypertensive disorders across the different study cohorts is noteworthy. In our study, only 9.4% and 1.9% of the women had diabetic and hypertensive disorders, respectively. In Rao’s cohort, 19.6% of women with TOLAC had gestational diabetes mellitus, and 2.6% had hypertension disorders [40]. Second, the sample sizes were relatively small and may have introduced bias when managing women with diabetes or hypertensive disorders.

One strength of this analysis was that the method of model development incorporated best practices, such as LASSO regression analysis for variable selection and the application of both internal and temporal validation techniques. The inclusion of decision curve analysis further enhances the utility of the model, facilitating informed counselling for women making decisions regarding TOLAC. Moreover, the overall chance of achieving VBAC once a TOLAC was undertaken and the total CS rate remained stable during the study period. The VBAC rate in our cohort was 81.4%, which was more than the 80% used to create the model. Using 81.4% as the VBAC rate yielded a sample size of 311. It was reassuring that our development set had 481 women, which was well above 311.

This study had some limitations. First, the retrospective nature of our study inherently carries limitations associated with the retrospective research design. Second, the demographic and clinical characteristics of the TOLAC population in the temporal validation set differed from those in the development set. These variations pose potential challenges by introducing factors that contribute to calibration drift and, consequently, a decline in both discrimination and calibration of the model to a certain extent. To mitigate calibration drift, we have minimized overfitting through LASSO and ensured data collection using consistent and standardized methods. Furthermore, it is essential to identify more appropriate predictive variables in future studies to update and optimize the model. Despite these challenges, the model maintained commendable predictive efficacy, suggesting its utility for application to new populations. The tertiary group had a relatively small sample size. Finally, in addition to maternal and fetal characteristics, another important predictor of VBAC is the health providers managing labor and the center women are laboring in, which cannot be generalized into a calculator. The study was conducted at a single academic medical center with high resources; thus, the generalizability of our findings is limited to centers with comparable medical resources. Additional large-scale, prospective, multicenter studies are required to clinically evaluate and validate the model developed in this study. Such follow-up studies are also needed to confirm whether or not the findings of this current study may be replicated in real-world clinical settings.

## 5. Conclusion

The improved predictive VBAC model revealed adequate performance such that women with a prior low transverse cesarean delivery who scored 0.7 or higher (in the model-derived probability score) would consider TOLAC, potentially leading to a reduction in maternal-neonatal morbidity.

## Supplementary Material

Supplemental Material

Supplemental Material

## Data Availability

The datasets analyzed in the current study are available from the corresponding author upon reasonable request.
