# Supplementary material for: Development and validation of an improved prediction model for vaginal birth after previous cesarean section: a retrospective cohort study of a Chinese population
Source: Ann Med. 2025 Jun 28;57(1):2523617. doi: 10.1080/07853890.2025.2523617 (PMC12207765; doi:10.1080/07853890.2025.2523617)
Supplement: Supplemental Material [file IANN_A_2523617_SM8075.docx]

Table S1 Delivery outcomes stratified by VBAC of the total cohort (n=720).

| Variables | total cohort (n=720) | VBAC (n=586) | failed TOLAC (n=134) | *P* value* |
| --- | --- | --- | --- | --- |
| macrosomia, % | 40 (5.6) | 28 (4.8) | 12 (9.0%) | 0.059 |
| maternal complications |  |  |  |  |
| postpartum hemorrhage, % | 35 (4.9) | 31 (5.3) | 4 (3.0) | 0.263 |
| postpartum blood loss ≥ 1500ml, % | 4 (0.6) | 2 (0.3) | 2 (1.5) | 0.159 |
| blood transfusions, % | 12 (1.7) | 9 (1.5) | 3 (2.2) | 0.842 |
| uterine rupture, % | 12 (1.7) | 1 (0.2) | 11 (8.2) | <0.001 |
| complete uterine rupture, % | 3 (0.4) | 0 | 3 (2.2) | 0.006 |
| incomplete uterine rupture, % | 9 (1.3) | 1 (0.2) | 8 (6.0) | <0.001 |
| 3rd and 4th perineal tears | 0 | 0 | 0 | - |
| hysterectomy, % | 0 | 0 | 0 | - |
| bladder injury, % | 1 (0.1) | 0 | 1 (0.7) | 0.186 |
| neonatal complications |  |  |  |  |
| umbilical artery blood gas pH ≤7.1, % | 2 (0.3) | 1 (0.2) | 1 (0.7) | 0.338 |
| 1 min Apgar score ≤7, % | 7 (1.0) | 2 (0.3) | 5 (3.7) | 0.020 |
| neonatal asphyxia, % | 5 (0.7) | 1 (0.2) | 4 (3.0) | 0.005 |
| NICU, % | 19 (2.6) | 14 (2.4) | 5 (3.7) | 0.565 |

TOLAC, trial of labor after cesarean; VBAC, vaginal birth after cesarean; NICU, neonatal intensive care unit. * Chi-square test or Fisher’s exact test as appropriate.
