# Supplementary material for: Development and validation of an improved prediction model for vaginal birth after previous cesarean section: a retrospective cohort study of a Chinese population
Source: Ann Med. 2025 Jun 28;57(1):2523617. doi: 10.1080/07853890.2025.2523617 (PMC12207765; doi:10.1080/07853890.2025.2523617)
Supplement: Supplemental Material [file IANN_A_2523617_SM8074.docx]

Table S2 Variable selection by LASSO regression in the minimal average error.

| Variables | Coefficients |
| --- | --- |
| maternal height | 0.0112 |
| newborn weight | -0.1444 |
| the ratio of weight gain to pre-pregnancy weight | -0.5271 |
| maternal age | -0.5911 |
| interval time of pregnancies | -0.3839 |
| spontaneous labor onset | 0.8424 |
| oxytocin administration | -1. 39458 |
| PROM | -0.2924 |
| labor analgesia | 1.1903 |
| vaginal delivery history | 0.3855 |
| λ value | 0.0086 |
| numbers of variables | 10 |

LASSO, least absolute shrinkage and selection operator; PROM, premature rupture of membranes.
